# A Unique Case of Bannwarth Syndrome in Early Disseminated Lyme Disease

**DOI:** 10.7759/cureus.14680

**Published:** 2021-04-25

**Authors:** Yetunde B Omotosho, Robin Sherchan, Grace W Ying, Maryna Shayuk

**Affiliations:** 1 Internal Medicine, Chicago Medical School Internal Medicine Residency Program at Northwestern Mchenry Hospital, McHenry, USA; 2 Internal Medicine, Chicago Medical School Internal Medicine Residency Program at Northwestern McHenry Hospital, McHenry, USA

**Keywords:** bannwarth syndrome, neuroborreliosis, lyme's disease, radiculoneuritis, tick-borne infections

## Abstract

Lyme borreliosis is a multisystem inflammatory disease caused by the spirochete *Borrelia burgdorferi* (*B. burgdorferi)* and transmitted through the *Ixodes* tick. Nervous system involvement is known as Lyme neuroborreliosis; it only occurs in disseminated Lyme disease and is manifested by the classic triad of meningitis, cranial neuritis, and radiculoneuritis. Timeline is a significant factor when staging Lyme manifestations. However, certain cases do not follow the typical presentation timelines described in most literature. We report a case of a 66-year-old male who presented with progressively worsening generalized body aches, severe fatigue, and new-onset urine retention for two weeks. Physical examination revealed severe pain with neck flexion and lying supine and diminished deep tendon reflexes bilaterally. Laboratory data revealed a positive Lyme immunoglobulin (Ig) M antibody with lymphocytic pleocytosis on lumbar puncture. He was treated with intravenous (IV) ceftriaxone for early disseminated Lyme disease. His radicular pain responded well to therapy, and he regained full bladder function. Bannwarth syndrome (BWS) is a term applied to the constellation of painful radiculoneuritis characterized as severe, burning, often dermatomal pain. In most cases, BWS affects the limbs, with only a few reported cases of sacral radiculitis causing neurogenic urinary dysfunction. Early recognition of this rare presentation associated with Lyme disease and treatment with antibiotics can prevent disease progression and detrimental neurological sequelae.

## Introduction

Lyme disease is an illness caused primarily by three pathogenic species of spirochete *Borrelia* (*B. burgdorferi*, *B. afzelii*, and *B. garinii*) and transmitted through the *Ixodes* tick. The principal causative agent of Lyme disease in the United States is *B. burgdorferi* [1]. Specifically, Lyme neuroborreliosis (LNB) is reported in 10-15% of Lyme disease cases in the United States [2]. Nervous system involvement starts during early disseminated Lyme disease secondary to meningeal seeding from spirochete spread. LNB is characterized by numerous clinical features, which often makes the diagnostic process challenging. However, lymphocytic meningitis, cranial neuropathy such as Bell’s palsy, and radiculoneuritis constitute the classic triad of early neurologic Lyme disease. BWS is an uncommon manifestation of neuroinvasive Lyme disease that has been reported in Europe. It is the most common manifestation of acute Lyme borreliosis among adults in Europe after erythema migrans [3]. This presentation, however, is likely underdiagnosed in the United States. BWS is characterized by a wide range of symptoms including radicular pain (100%), sleep disturbances (75.3%), headache (46.8%), fatigue (44.2%), malaise (39%), paresthesia (32.5%), peripheral nerve palsy (36.4%), meningeal signs (19.5%), and paresis (7.8%) [3]. BWS manifesting as sacral radiculitis presents with urinary symptoms, including urine retention [4]. The onset of symptoms can vary from weeks to months after exposure.

## Case presentation

We present a case of a 66-year-old male with no significant medical history who presented to the emergency room with a two-week history of generalized myalgia, fatigue, and severe neck pain. His symptoms started two days after doing some extensive yard work, during which time he removed two ticks attached to his skin. The duration and depth of the tick attachment were unknown to the patient. He first noticed a dull mid-back pain radiating down his neck and exacerbated by neck flexion. He subsequently noted intermittent dull headache with scalp tenderness and neck stiffness. His pain then radiated down his entire spine into his upper and lower extremities, leading to right arm weakness and new urine retention onset. On day 6 of his illness, he notified his primary physician, who prescribed an unspecified antibiotic for suspected walking pneumonia and analgesics, with no improvement of symptoms.

His physical examination revealed stable vitals on admission, absence of skin rash or erythema, a pain scale of 8/10, with paraspinal tenderness, and diminished deep tendon reflexes bilaterally. Laboratory data were significant for a white blood cell count of 12 k/uL, C-reactive protein of 8.8 mg/L, sedimentation rate of 100 mm/h, and creatinine kinase of 27 units/L. Other tests ruled out anaplasmosis and ehrlichiosis. Autoimmune workup including antinuclear antibody (ANA), aldolase, serum immunoglobulins, and protein electrophoresis was negative. Rheumatoid factor was also negative. Lyme serology was positive at 8.42 (<0.90 is negative, 0.90-1.09 is equivocal, >1.09 is positive). Western blot confirmed three of three positive immunoglobulin M (IgM) immunoblots and 1 of 10 positive immunoglobulin G (IgG) immunoblots, which confirmed the infection's early stage (Table 1).

**Table 1:** Lyme disease antibodies (IgG, IgM), immunoblot showing 3/3 positive IgM bands, compatible with early disease. As per CDC criteria, a Lyme disease IgG Immunoblot must show reactivity to at least 5 of 10 specific borrelial proteins to be considered positive; similarly, a positive Lyme disease IgM immunoblot requires reactivity to two of three specific borrelial proteins. AB, antibodies; IgG, immunoglobulin G; IgM, immunoglobulin M; CDC, Centers for Disease Control and Prevention

Name	Value	Reference Range
Lyme disease AB (IgG), blot	Negative	Negative
18-KD (IgG) band	Reactive	
23-KD (IgG) band	Non-reactive	
28-KD (IgG) band	Non-reactive	
30-KD (IgG) band	Non-reactive	
39-KD (IgG) band	Non-reactive	
41-KD (IgG) band	Non-reactive	
45-KD (IgG) band	Non-reactive	
58-KD (IgG) band	Non-reactive	
66-KD (IgG) band	Non-reactive	
93-KD (IgG) band	Non-reactive	
Lyme disease AB (IgM), blot	Positive	Negative
23-KD (IgM) band	Reactive	
39-KD (IgM) band	Reactive	
41-KD (IgM) band	Reactive	

**Figure 1:**
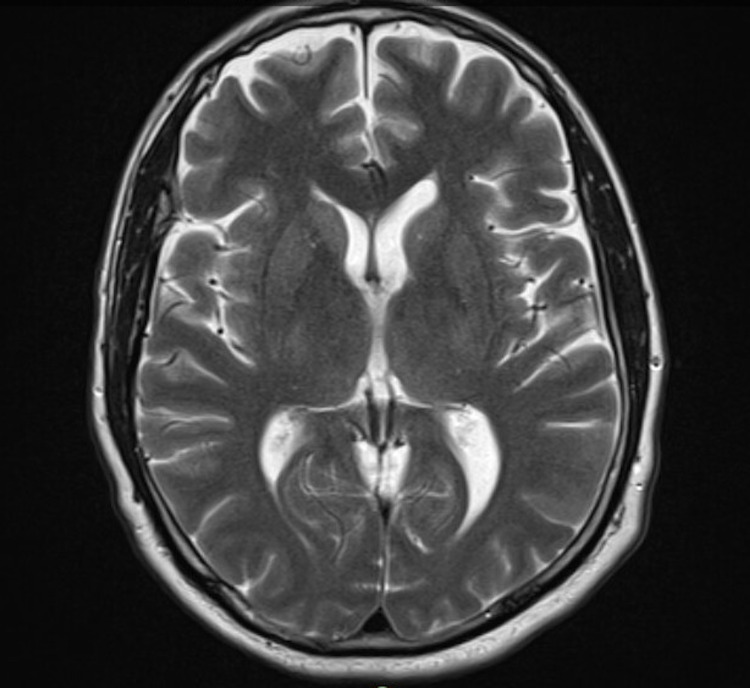
Normal brain MRI.

Extensive imaging studies, including magnetic resonance imaging (MRI) of the brain (Figure 1) and MRI of the cervical/thoracic/lumbar spine, did not reveal any abnormalities.

Lumbar puncture was obtained, with cerebrospinal fluid (CSF) analysis revealing lymphocytic pleocytosis (CSF lymphocytes 73%, CSF monocytes 26%, CSF neutrophils 1%) and positive Lyme titers (Table 2).

**Table 2:** CSF fluid analysis showing lymphocytic pleocytosis and elevated CSF proteins with normal CSF glucose. *High. **Very high. CSF, cerebrospinal fluid

Component	Latest Reference Range and Units	Values
Total cells counted, CSF		100
Neutrophils, CSF	≤7%	1
Lymphocytes, CSF	<35%	73 (H)*
Monocytes, CSF	<90 %	26
Smear review		
Glucose, CSF	Based on documented legal sex: 40-70 mg/dL	54
Protein, CSF	15-45 mg/dL	118 (HH)**

The patient was treated with IV ceftriaxone and doxycycline. Doxycycline was discontinued after antibody titers to anaplasmosis and ehrlichiosis came back negative. Our patient responded to treatment, and his symptoms improved with complete resolution of his urinary retention, evidenced by the amount of urine spontaneously voided. He was discharged in stable condition. He completed 21 days of treatment with IV ceftriaxone and was fully recovered at his follow-up visit three weeks later.

## Discussion

Lyme disease is the most common tick-borne infection in the United States [5]. The Centers for Disease Control and Prevention (CDC) estimates about 300,000 new cases annually [6]. It is most prevalent in the Northeast, mid-Atlantic, and upper Midwest regions, but cases have also been reported in certain Pacific Coast areas [7]. Nervous system disease occurs in 10% to 15% of untreated patients [1]. Lyme disease has three stages: early localized, early disseminated, and late/chronic disease. Although all age groups are affected by Lyme disease, the distribution of cases is markedly bimodal, with peaks among children 5-14 years old and adults 45-55 years old [2]. Nervous system involvement can be divided into three distinct but potentially overlapping categories, including (1) extra parenchymal central nervous system involvement that mainly occur in children, manifesting as meningitis or pseudotumor cerebi-like picture; (2) unifocal or multifocal inflammation of the peripheral nerves; and (3) unifocal or multifocal inflammation of the brain and spinal cord parenchyma. These disorders can occur individually or in combination [8]. *Borrelia burgdorferi* enters the skin through the site of the tick bite. Within 3 to 32 days, the organisms can migrate in the skin around the bite area, spread through the lymphatics to cause regional adenopathy, or disperse in the blood to various organs. Neuroborreliosis begins during early disseminated Lyme disease when the spread of spirochetes can result in meningeal seeding [9]. An inflammatory reaction (erythema migrans) occurs at the initial stage of infection before a significant antibody response occurs (serologic conversion). Early LNB often occurs three to six weeks after the infection and most frequently presents as lymphocytic meningoradiculoneuritis [10].

Garin and Bujadoux were the first to report painful radiculopathy in the 1920s. It has since been referred to as Garin-Bujadoux-Bannwarth’s syndrome, which has gained much attention, particularly in the European literature [3]. Despite controversies regarding its incidence in the United States, the condition does occur but is often misdiagnosed [8]. BWS is characterized by painful radiculopathy, neuropathy, varying degrees of motor weakness, facial nerve palsy, and cerebrospinal fluid pleocytosis [11]. BWS typically manifests itself with severe zoster-like segmental pain that is worse at night. The pain has a burning, stabbing, biting, or tearing character and usually responds poorly to all common analgesics. Around 75% of patients with BWS develop neurological deficits in one to four weeks, generally in the form of flaccid paralysis or segmental sensory disturbance. Around 60% of them have cranial nerve deficits, and within this population, more than 80% have facial nerve involvement, leading to typical peripheral facial palsy [12].

There are a few reported cases of neurogenic urinary tract dysfunction from *B. burgdorferi* induced sacral radiculitis. The urinary tract may be affected in two ways: first, with the influence of the radiculitis on innervating fibers leading to weakness of the detrusor muscle, and, second, detrusor weakness as a result of direct invasion of the spirochetes into the bladder wall. Both mechanisms can result in detrusor hyperreflexia, detrusor areflexia, or detrusor-sphincter dyssynergia [13]. As uniquely reported in this case, our patient started showing signs of nervous system involvement within two days of exposure to *B. burgdorferi*, manifesting as severe radiculopathy, upper extremity weakness, and urinary dysfunction. All of these findings are pathognomonic for BWS. The positive IgM antibody signified early infection, and the quick response to ceftriaxone also supported the diagnosis.

The diagnosis of Lyme disease can be made based on the cutaneous findings alone or by serologic testing. The standard approach to serodiagnosis is a two-tier process, starting with enzyme-linked immunosorbent assay (ELISA), a method that estimates the total antibodies in the patient’s serum reacting with the causative organism. Positive tests are confirmed using a method that detects the particular bacterial protein to which the antibodies bind, referred to as Western blot [8]. An early phase of the disease, often between the third and sixth week, produces a robust IgM response. The presence of two out of three IgM antibody peptides is considered positive. After four to six weeks of disease, the immune response will be switched to IgG production. A positive result has been statistically shown to require at least 5 of 10 IgG bands. Of note, IgM blots become uninterpretable outside six weeks of infection, as this stage of the disease should rather have IgG seropositivity [8].

The decision to assess CSF in patients with facial nerve palsy or BWS is subject to debate [14]. A reasonable approach is to perform lumbar puncture only if the diagnosis is unclear, and antibiotics should be administered to patients with recent exposure, an appropriate clinical presentation, and positive serology [15]. Although CSF cultures are rarely positive in Lyme disease patients, lymphocytic pleocytosis is present in over 80% of cases [16]. One of the most important concepts to understand is that a positive Lyme disease serology in CSF does not mean that the patient has LNB [17]. It could represent evidence of a previous infection or simply reflecting potential leakage of serum antibodies across the blood-brain barrier. IgG and IgM antibodies may persist in the CSF long after adequate treatment and in the absence of clinical evidence of active neurologic disease [16].

In patients with peripheral neuropathy, electrophysiology assessments such as electromyography and nerve conduction studies can be helpful. In such patients, these studies typically reveal findings consistent with a patchy axonal polyneuropathy (i.e., mononeuropathy multiplex) [18]. In patients with non-focal cognitive difficulties, memory concerns, psychiatric issues, or unclear evidence to suggest Lyme meningitis or encephalomyelitis, brain imaging is rarely abnormal and of low diagnostic value. In BWS cases presenting with a neurogenic bladder like in our patient, it is reasonable to obtain an MRI of the lumbar spine to rule out other possible spinal etiologies. However, this approach is usually not warranted and also of low diagnostic value.

Early Lyme disease, including cardiac and neurological involvement, can be treated with a 14-day course (range: 10-21 days) of doxycycline [7]. Cases of Lyme meningitis, encephalitis, radiculopathies/BWS, carditis with symptoms, and arthritis unresponsive to initial oral antibiotics require a 14-day (range: 10-28 days) course of IV antibiotic therapy, typically ceftriaxone [19]. Doxycycline has the advantage of being effective for treating anaplasmosis, which may coincide simultaneously with early Lyme disease.

## Conclusions

Although once rare, BWS is becoming increasingly prevalent in the United States. The constellation of neurological symptoms, particularly when associated with a recent or suspected tick bite in an endemic region, should prompt thorough evaluation for LNB and assessment for BWS. Physicians need to be aware of the rare neurological manifestations of LNB, especially those with a non-pathognomonic timeline at presentation, as shown in our case. Prompt diagnosis and treatment with antibiotics can reduce unnecessary imaging, patient anxiety, and, most importantly, avert debilitating complications.

## References

[1] Schwartz AM, Hinckley AF, Mead PS, Hook SA, Kugeler KJ (2017). Surveillance for Lyme disease - United States, 2008-2015. MMWR Surveill Summ.

[2] Centers for Disease Control and Prevention (2004). Lyme disease--United States, 2001-2002. MMWR Morb Mortal Wkly Rep.

[3] Hansen K, Lebech AM (1992). The clinical and epidemiological profile of lyme neuroborreliosis in Denmark 1985-1990: a prospective study of 187 patients with Borrelia burgdorferi specific intrathecal antibody production. Brain.

[4] Chancellor Michael B, Dato Virginia M, Yang John Y (1990). Lyme disease presenting as urinary retention. J Urol.

[5] Adams DA, Thomas KR, Jajosky RA (2016). Summary of notifiable infectious diseases and conditions - United States, 2014. MMWR Morb Mortal Wkly Rep.

[6] Kuehn BM (2013). CDC estimates 300,000 US cases of Lyme disease annually. JAMA.

[7] Bender PD, Ilgen JS (2018). Early disseminated Lyme disease. BMJ Case Rep.

[8] (2021). A Neurologist's View of Lyme Disease and Other Tick-Borne Infections. https://www.medscape.com/viewarticle/919614.

[9] Luft BJ, Steinman CR, Neimark HC (1992). Invasion of the central nervous system by Borrelia burgdorferi in acute disseminated infection. JAMA.

[10] Hansen K, Crone C, Kristoferitsch W (2013). Lyme neuroborreliosis. Handb Clin Neurol.

[11] Shah A, O'Horo JC, Wilson JW, Granger D, Theel ES (2018). An unusual cluster of neuroinvasive Lyme disease cases presenting with Bannwarth syndrome in the Midwest United States. Open Forum Infect Dis.

[12] Rauer S, Kastenbauer S, Fingerle V, Hunfeld KP, Huppertz HI, Dersch R (2018). Lyme neuroborreliosis. Dtsch Arztebl Int.

[13] Chancellor MB, McGinnis DE, Shenot PJ, Kiilholma P, Hirsch IH (1993). Urinary dysfunction in Lyme disease. J Urol.

[14] Rupprecht TA, Pfister HW (2009). What are the indications for lumbar puncture in patients with Lyme disease?. Curr Probl Dermatol.

[15] Halperin JJ, Shapiro ED, Logigian E (2007). Practice parameter: treatment of nervous system Lyme disease (an evidence-based review): report of the Quality Standards Subcommittee of the American Academy of Neurology. Neurology.

[16] (2021). Lyme Disease: Practice Essentials, Background, Etiology. https://emedicine.medscape.com/article/330178-overview#a3.

[17] Roos KL, Berger JR (2007). Is the presence of antibodies in CSF sufficient to make a definitive diagnosis of Lyme disease?. Neurology.

[18] Logigian EL, Steere AC (1992). Clinical and electrophysiologic findings in chronic neuropathy of Lyme disease. Neurology.

[19] Wormser GP, Dattwyler RJ, Shapiro ED (2006). The clinical assessment, treatment, and prevention of lyme disease, human granulocytic anaplasmosis, and babesiosis: clinical practice guidelines by the Infectious Diseases Society of America. Clin Infect Dis.

